# Renal Function and Efficacy of Remote Ischemic Conditioning in Acute Moderate Ischemic Stroke: A Post Hoc Analysis of RICAMIS Trial

**DOI:** 10.1002/brb3.70831

**Published:** 2025-10-07

**Authors:** Xiao‐Yi He, Yu Cui, Jia‐Qi Wang, Lu Wang, Hui‐Sheng Chen

**Affiliations:** ^1^ Department of Neurology General Hospital of Northern Theater Command Shenyang China; ^2^ Dalian Medical University Dalian China; ^3^ Department of Neurology Affiliated Zhongshan Hospital of Dalian University Dalian China

**Keywords:** acute ischemic stroke, functional outcome, remote ischemic conditioning, renal function

## Abstract

**Background:**

Emerging evidence supports the therapeutic potential of remote ischemic conditioning (RIC) in acute stroke management. This post hoc analysis aims to elucidate the relationship between renal function and the efficacy of RIC.

**Methods:**

This post hoc analysis utilized the complete case dataset from the RICAMIS trial, enrolling acute ischemic stroke patients with documented renal function within 48 h of symptom onset. Participants were categorized into three groups based on their estimated glomerular filtration rate (eGFR) at admission: normal (≥ 90 mL/min/1.73 m^2^), mildly impaired (60–89 mL/min/1.73m^2^), and severely impaired renal function (< 60 mL/min/1.73 m^2^). The primary outcome was an excellent functional outcome, defined as a modified Rankin Scale score of 0–1 at 90 days. We examined the association between RIC and functional outcomes within each renal function group, as well as the interaction between RIC efficacy and renal function, in comparison to patients receiving standard care.

**Results:**

Of the 1652 patients evaluated, 941 had normal renal function, 605 had mildly impaired renal function, and 106 had moderate to severely impaired renal function. In comparison to the control group, RIC was associated with a higher probability of achieving the primary outcome in patients with normal renal function (69% vs. 64%) and those with mildly impaired renal function (66.7% vs. 59.7%). However, this association was not observed in patients with moderate to severely impaired renal function (64.1% vs. 66%, *p* = 0.428). There was no significant interaction between renal function and the efficacy of RIC (adjusted *p* value = 0.970).

**Conclusion:**

Compared with moderate to severely impaired renal function, RIC is associated with an increased likelihood of excellent functional outcome at 90 days in patients with normal and mildly impaired renal function.

**Trial Registration:**

ClinicalTrials.gov identifier: NCT03740971

## Graphical abstract

• It remains unclear as to whether renal function is associated with remote ischemic conditioning (RIC) efficacy in acute ischemic stroke.
• Compared with moderate to severely impaired renal function, RIC is associated with an increased likelihood of excellent functional outcome at 90 days in patients with normal and mildly impaired renal function.
• This study suggests that renal function may be associated with RIC efficacy.

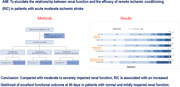

## 1 Introduction

There have been many studies investigating the neuroprotective effect of remote ischemic conditioning (RIC) (Hess et al. 2015; Xu et al. 2023), but the study results have been mixed (Pico et al. 2020; England et al. 2017; Hou et al. 2022; Li et al. 2024). Recently, the Remote Ischemic Conditioning for Acute Moderate Ischemic Stroke (RICAMIS) randomized trial provided robust evidence that RIC can improve clinical outcomes in patients with acute moderate ischemic stroke (Chen et al. 2022). Over one‐third of patients with ischemic stroke concomitantly suffer from chronic kidney disease (CKD) (Ovbiagele et al. 2014). A large meta‐analysis of 83 studies that explored the relationship between estimated glomerular filtration rate (eGFR) and stroke risk found that as eGFR fell, the risk of stroke increased in a dose‐response fashion (Masson et al. 2015). Previous studies have shown that kidney disease is associated with greater neurological deficit following ischemic stroke, poor functional outcome, and greater mortality (Ovbiagele et al. 2009; Kumai et al. 2012; El Husseini et al. 2017, 2018; Alqahtani et al. 2018; Synhaeve et al. 2016; Ovbiagele et al. 2011). On the one hand, renal function was found to be associated with the treatment effect on stroke, including antiplatelet treatment (Wang et al. 2022), intravenous thrombolysis (Carr et al. 2017), and endovascular treatment (Xiao et al. 2020). On the other hand, RIC has been found to be an effective tool to protect certain organs, such as the kidney (Giannopoulos et al. 2017).

Based on the above discussion, we hypothesized that renal function may affect the efficacy of RIC after stroke.

In this context, we performed this secondary analysis of the RICAMIS trial to investigate this issue.

## 2 Methods

### 2.1 Study Design and Participants

Details on the design, protocol, and primary outcome analysis of RICAMIS have been published previously (Chen et al. 2022). Briefly, the RICAMIS study was a multicenter, open‐label, blinded‐endpoint, randomized clinical trial conducted between December 26, 2018, and April 19, 2021, aiming to explore the benefit of 2 weeks of RIC in patients with acute moderate ischemic stroke. Eligible patients were adults aged 18 years or older with acute moderate ischemic stroke presenting within 48 h of onset at the time of randomization (baseline National Institutes of Health Stroke Scale [NIHSS] scores, 6–16) who had been functioning independently in the community before a stroke (modified Rankin Scale [mRS] scores, 0–1). The main exclusion criteria were patients who received intravenous thrombolysis or endovascular therapy, had any contraindication for RIC, had cardiogenic embolism, or patients without eGFR measurement due to a lack of creatinine examination. The study was performed in accordance with the Declaration of Helsinki and relevant guidelines, and approved by the ethics committee of the General Hospital of Northern Theater Command (ethics approval ID: k2018[43]). All patients or their legally authorized representatives signed a written informed consent form before entering the study.

### 2.2 Procedures

All patients were allocated to treatment with either RIC treatment in addition to guideline‐recommended treatment (such as antiplatelet, anticoagulant, or statins) or only guideline‐recommended treatment. According to the National Kidney Foundation Kidney Disease Outcomes Quality Initiative (NKF‐KDOQI) guidelines (Levey et al. 2003; Stevens et al. 2013), patients were divided into three groups: normal renal function group (eGFR ≥ 90 mL/ min /1.73 m^2^), mildly impaired renal function group (eGFR: 60 to 89 mL/ min /1.73 m^2^), and moderate to severely impaired renal function group (eGFR < 60 mL /min/1.73 m^2^). Considering the small sample size of patients with moderate to severely impaired renal function, these patients were pooled into one group as moderate to severely impaired renal function. Each group was subdivided into RIC and control treatment groups. RIC treatment was initiated within 48 h of symptom onset, which was performed by five cycles of cuff inflation (200 mmHg for 5 min) and deflation (for 5 min), for a total procedure time of 50 min, twice daily for 10–14 days. Further details of RIC treatment were described in a previous report (Chen et al. 2022). Neurologic status, measured using the NIHSS score, was evaluated at admission, 7 days, and 12 days after randomization. Follow‐up data including the assessment of the patient's prognosis were collected 90 days after randomization.

### 2.3 Calculation of eGFR

Venous blood samples were obtained before randomization. The eGFR was calculated using the Chronic Kidney Disease Epidemiology Collaboration creatinine equation (CKD‐EPI) (Levey et al. 2009): eGFR = 141×min (SCr/*k*,1)^α^× max (SCr/*k*,1)^−1.209^×0.993^Age^×1.018 (if the patient is female), where SCr is serum creatinine, *k* is 0.7 for female patients and 0.9 for male patients,α is −0.329 for female patients and −0.411 for male patients, min is the minimum of SCr/*k* or 1, and max indicates the maximum of SCr/*k* or 1. The CKD‐EPI China equation was calculated with a coefficient of 1.1 (Teo et al. 2011).

### 2.4 Study Outcomes

The primary outcome was excellent functional outcome, defined as an mRS score of 0–1 at 90 days. The secondary outcomes included favorable functional outcome at 90 days (mRS score of 0–2), the occurrence of early neurological deterioration compared with baseline at 7 days, defined as an increase of more than 2 points on the NIHSS, but not as a result of cerebral hemorrhage, the occurrence of stroke‐associated pneumonia at 12 days, change in NIHSS score compared with baseline at 12 days, time from randomization to occurrence of stroke or other vascular events at 90 days, and the occurrence of death within 90 days.

### 2.5 Statistical Analysis

For baseline characteristics of eligible patients, we summarized continuous variables with normal distribution as mean (standard deviation) and categorical variables as frequencies (percentages). For continuous variables, the *t*‐test was used for comparison between groups. For categorical variables, the chi‐square test was used for comparison between groups.

First, the probability of excellent functional outcomes at 90 days was calculated in patients receiving RIC treatment as an adjunct to usual care and only usual care through binary logistic regression analysis. The probability curves with their 95% confidence intervals (CIs) stratified according to probability and renal function in each treatment group were drawn.

Second, we compared the efficacy of RIC treatment with usual care in each renal function group. The primary analysis of the current study was adjusted to account for baseline variables that had a difference between groups with *p* value < 0.05. Binary logistic regression was performed to evaluate the association between RIC treatment and outcomes, such as excellent functional outcomes and favorable functional outcomes at 90 days, which generated an odds ratio (OR) of outcomes between RIC and control groups together with two‐sided 95% CIs and *p* values. Cox regression analysis was performed to investigate the association between RIC treatment and outcomes, such as time from randomization to occurrence of stroke or other vascular events at 90 days and death at 90 days, which generated a hazard ratio (HR) with two‐sided 95% CI and *p* values. Missing data of covariates in the adjusted analyses were imputed through simple imputation.

Third, the assessments of the association between renal function and the effect of RIC treatment on primary and secondary outcomes were conducted by binary logistic regression with the treatment, renal function groups, and their interaction term as independent variables, and the *p* value presented for the interaction term. The adjusted interactions were conducted through including imbalance baseline variables between renal function groups with *p* value < 0.05 in the model.

All analyses presented were exploratory, and all *p* values were nominal. Two‐sided *p* values < 0.05 were considered significant. SPSS software version 26.0 (IBM) and R software version 4.1.0 (R Foundation for Statistical Computing) was used for statistical analysis.

## 3 Results

### 3.1 Baseline Characteristics

**FIGURE 1:**
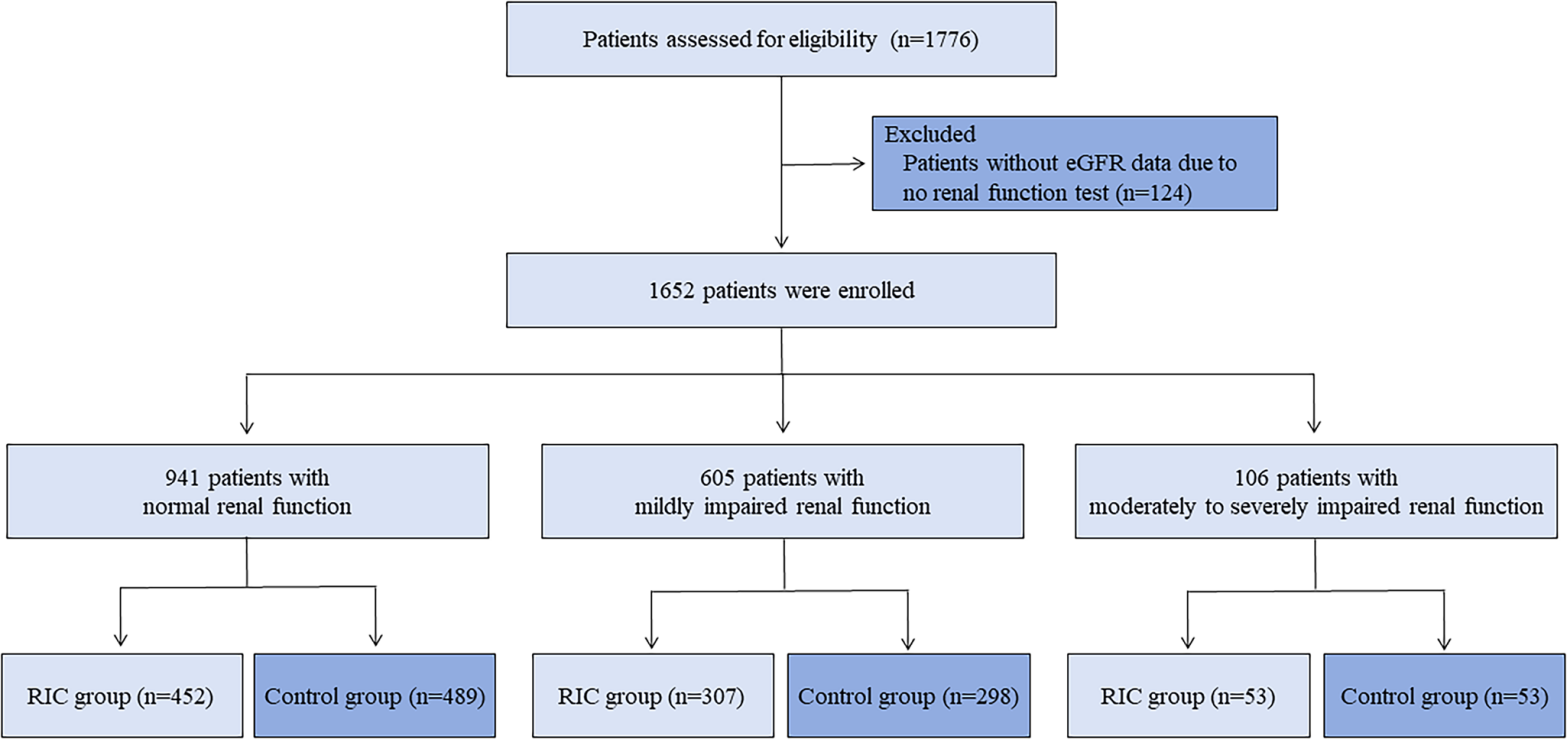
Flow chart of the study.

After excluding patients without eGFR data (*n* = 124), 1652 patients were included in this study from the full analysis set, including 941 (57.0%) with normal renal function, 605 (36.6%) with mildly impaired renal function, and 106 (6.4%) with moderate to severely impaired renal function (Figure 1). The baseline characteristics of patients were unbalanced by age, sex, smoking consumption, alcohol consumption, hypertension, and diabetes among the three renal function groups (Table 1). The baseline characteristics between the RIC and control groups across the three renal function groups were well balanced except for sex, smoking consumption, alcohol consumption, and hypertension in some subgroups (Table 2).

**TABLE 1:** Baseline characteristics of patients in each eGFR category.

Characteristics	Normal renal function (*n*= 941)	Mildly impaired renal function (*n* = 605)	Moderate to severely impaired renal function (*n* = 106)	*p* value
Age, years	61.3 (8.9)	70.7 (9.1)	71.5 (10.8)	<0.001
Sex				0.003
Male	645 (68.5)	383 (63.3)	57 (53.8)	
Female	296 (31.5)	222 (36.7)	49 (46.2)	
Body mass index^a^	24.5 (3.7)	24.2 (4.3)	24.3 (3.5)	0.516
Current smoker	287/917 (31.3)	159/581 (27.4)	21/101 (20.8)	0.042
Current drinker^b^	139/925 (15.0)	74/590 (12.5)	6/101 (5.9)	0.027
Comorbidities				
Hypertension	569/929 (61.2)	367/597 (61.5)	82/105 (78.1)	0.003
Diabetes	243/936 (26.0)	124/604 (20.5)	33/106 (31.1)	0.013
Previous stroke	286/938 (30.5)	208/599 (34.7)	41/104 (39.4)	0.069
Previous TIA	11/939 (1.2)	8/605 (1.3)	1/104 (1.0)	0.129
Blood pressure at randomization, mm Hg				
Systolic	150.5 (17.7)	152.4 (19.3)	154.6 (20.3)	0.032
> 140	613 (65.1)	401 (66.3)	79 (74.5)	0.153
Diastolic	89.0 (11.1)	88.4 (11.1)	88.6 (11.6)	0.588
> 90	343 (36.5)	217 (35.9)	42 (40.0)	0.760
Blood glucose, mmol/L	7.7 (3.5)	7.0 (2.8)	7.2 (3.0)	<0.001
> 7.0	343/857 (40.0)	183/555 (33.0)	32/91 (35.2)	0.026
Baseline NIHSS score^c^	7 (6–10)	7 (6–9)	7 (6–9)	
Estimated premorbid function (mRS score)				0.133
mRS 0	721 (76.6)	442 (73.1)	74 (69.8)	
mRS 1	220 (23.4)	163 (26.9)	32 (30.2)	
Presumed stroke cause^d^				0.183
LAA	265/939 (28.2)	183/605 (30.2)	39/106 (36.8)	
CE	7/939 (0.7)	10/605 (1.7)	1/106 (0.9)	
SAO	160/939 (17.0)	93/605 (15.4)	11/106 (10.4)	
ODC	9/939 (1.0)	10/605 (1.7)	0	
UND	498/939 (53.0)	309/605 (51.1)	55/106 (51.9)	

*Note*: Data were expressed as mean (SD) or No./total (%).

Abbreviations: CE, cardioembolic; LAA, large artery atherosclerosis; mRS, modified Rankin scale; NIHSS, National Institutes of Health Stroke Scale; ODC, other determined cause; UND, undetermined cause; SAO, small artery occlusion; TIA, transient ischemic attack.

^a^ Calculated as weight in kilograms divided by height in meters squared.

^b^ Current drinker means consuming alcohol at least once a week within 1 year before onset of the disease and consuming alcohol continuously for more than 1 year.

^c^ Patients with NIHSS scores of 6–16 were eligible for this study; NIHSS scores range from 0 to 42, with higher scores indicating more severe neurologic deficit.

^d^ The presumed stroke cause was classified according to the Trial of Org 10172 in Acute Stroke Treatment (TOAST) classification system.

**TABLE 2:** Baseline characteristics of patients between treatment groups in each eGFR category.

Characteristics	Normal renal function (*n*= 941)			Mildly impaired renal function (*n* = 605)			Moderate to severely impaired renal function (*n* = 106)		
	RIC (*n* = 452)	Control (*n* = 489)	*p* value	RIC (*n* = 307)	Control (*n* = 298)	*p* value	RIC (*n* = 53)	Control (*n* = 53)	*p* value
Age, years	61.0 (8.8)	61.6 (9.0)	0.248	71.0	70.4 (8.6)	0.390	70.1 (11.2)	73.0 (10.4)	0.171
Sex			0.385			0.037			0.846
Male	316 (69.9)	329 (67.2)		182 (59.3)	201 (67.4)		29 (54.7)	28 (52.8)	
Female	136 (30.0)	160 (32.7)		125 (40.7)	97 (32.5)		24 (45.3)	25 (47.2)	
Body mass index^a^	24.5 (4.4)	24.4 (2.8)	0.599	24.5 (5.2)	24.0 (3.0)	0.135	24.5 (3.5)	24.0 (3.5)	0.432
Current smoker	153/443 (34.5)	134/474 (28.3)	0.041	82/295 (27.8)	77/286 (27.0)	0.874	9/51 (17.6)	12/50 (24.0)	0.432
Current drinker^b^	80/446 (17.9)	59/479 (12.3)	0.017	41/300 (13.7)	33/290 (11.4)	0.402	5/52 (9.6)	1/49 (2.0)	0.206
Comorbidities									
Hypertension	267/449 (59.4)	302/480 (62.9)	0.281	191/301 (63.5)	176/296 (59.4)	0.316	46/52 (88.5)	36/53 (67.9)	0.011
Diabetes	114/452 (25.2)	129/484 (26.7)	0.618	62/306 (20.3)	62/298 (20.8)	0.869	17/53 (32.1)	16/53 (30.2)	0.834
Previous stroke	138/451 (30.6)	148/487 (30.4)	0.945	105/304 (34.5)	103/295 (34.9)	0.923	20/52 (38.5)	21/52 (40.4)	0.923
Previous TIA	5/450 (1.1)	6/489 (1.2)	0.869	5/307 (1.6)	3/298 (1.0)	0.503	0/53 (0)	1/51 (2)	0.314
Blood pressure at randomization, mmHg									
Systolic	150.2 (17.5)	150.9 (17.8)	0.568	151.4 (19.0)	153.4 (19.5)	0.193	156.1 (19.9)	153.2 (20.8)	0.459
>140	297 (65.7)	316 (64.6)	0.727	195 (63.5)	206 (69.1)	0.145	41 (77.4)	38 (71.7)	0.504
Diastolic	88.6 (11.4)	89.3 (11.2)	0.305	88.6 (11.2)	89.0 (11.4)	0.468	89.2 (11.2)	87.9 (11.9)	0.551
>90	161 (35.6)	182 (37.2)	0.611	99 (32.2)	118 (39.6)	0.060	23 (43.4)	19 (35.8)	0.427
Blood glucose, mmol/L	7.6 (3.4)	7.8 (3.5)	0.361	7.0 (2.7)	7.1 (2.8)	0.956	7.0 (2.5)	7.3 (3.5)	0.547
>7.0	150/411 (36.5)	193/446 (43.3)	0.043	94/283 (33.2)	89/272 (32.7)	0.901	16/46 (34.8)	16/45 (35.6)	0.938
Baseline NIHSS score^c^	7 (6–9)	7 (6–9)		7 (6–9)	7 (6–9)		7 (6–10)	6 (6–10)	
Estimated premorbid function (mRS score)			0.960			0.332			0.204
mRS 0	346 (76.5)	375 (76.6)		219 (71.3)	223 (74.8)		40 (75.5)	34 (64.2)	
mRS 1	106 (23.4)	114 (23.3)		88 (28.7)	75 (25.2)		13 (24.5)	19 (35.8)	
Presumed stroke cause ^d^			0.017			0.083			0.204
LAA	118/451 (26.2)	147/488 (30.1)		88/307 (28.7)	95/298 (31.8)		14/53 (26.4)	25/53 (47.2)	
CE	1/451 (0.2)	6/488 (1.2)		7/307 (2.3)	3/298 (1.0)		0	1/53 (1.9)	
SAO	67/451 (14.9)	93/488 (19.1)		40/307 (13.0)	53/298 (17.7)		8/53 (15.1)	3/53 (5.7)	
ODC	3/451 (0.67)	6/488 (1.2)		8/307 (2.6)	2/298 (0.6)		0	0	
UND	262/451 (58.1)	236/488 (48.4)		164/307 (53.4)	145/298 (48.6)		31/53 (58.5)	24/53 (45.3)	

*Note*: Data were expressed as mean (SD) or No./total (%).

Abbreviations: CE, cardioembolic; LAA, large artery atherosclerosis; mRS, modified Rankin scale; NIHSS, National Institutes of Health Stroke Scale; ODC, other determined cause; UND, undetermined cause; RIC, remote ischemic conditioning; SAO, small artery occlusion; TIA, transient ischemic attack.

^a^ Calculated as weight in kilograms divided by height in meters squared.

^b^ Current drinker means consuming alcohol at least once a week within 1 year before onset of the disease and consuming alcohol continuously for more than 1 year.

^c^ Patients with NIHSS scores of 6–16 were eligible for this study; NIHSS scores range from 0 to 42, with higher scores indicating more severe neurologic deficit.

^d^ The presumed stroke cause was classified according to the Trial of Org 10172 in Acute Stroke Treatment (TOAST) classification system.

**FIGURE 2:**
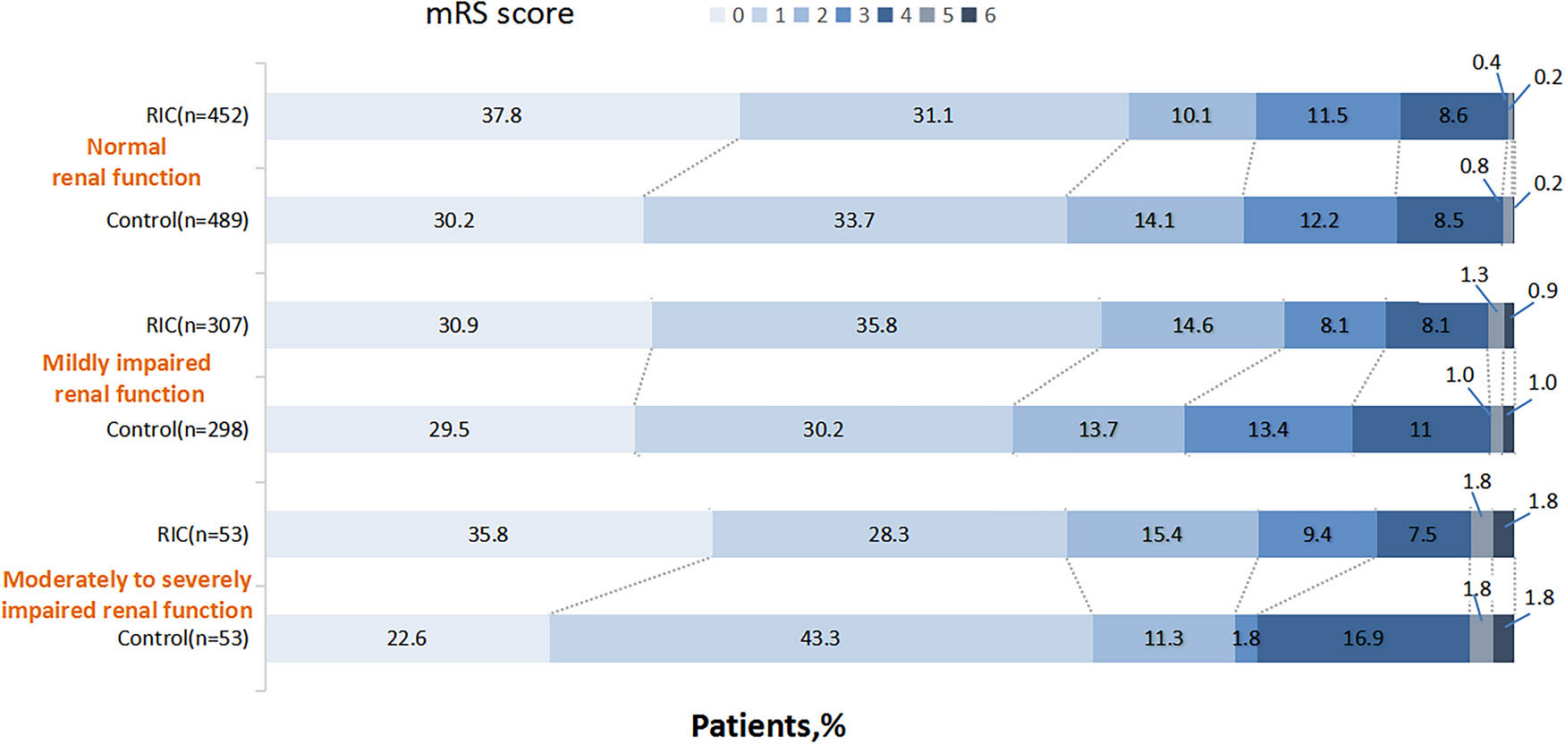
The relationship between the probability of modified Rankin Scale (mRS) score 0 to 1 and estimated glomerular filtration rate (eGFR).

Table 3 shows the association of RIC versus control with clinical outcomes stratified by eGFR category. Compared with the control group, a higher proportion of excellent function outcome (mRS 0–1) was associated with RIC treatment in patients with mildly impaired renal function (adjusted OR  =  1.382 [95% CI 0.990–1.929], *p* = 0.057), and patients with normal renal function (adjusted OR  =  1.245 [95% CI 0.947–1.635], *p* = 0.116), but not in patients with moderate to severely impaired renal function (adjusted OR  =  0.707 [95% CI 0.301–1.664], *p* = 0.428). Similar results were found in the distribution of mRS scores 90 days after treatment in patients with different renal functions (Figure 2).

**TABLE 3:** Association of RIC versus control with clinical outcomes stratified by eGFR category.

Outcome	Renal function impairment	RIC	Control	Treatment difference metric	*p* value	Adjusted^a^ treatment difference metric	Adjusted *p* value	Adjusted^b^ *P* interaction
mRS score of 0 to 1 at 90 d^c^	None	312 (69.0)	313 (64.0)	1.253 (0.955–1.644)	0.104	1.245 (0.947–1.635)	0.116	0.970
	Mild	205 (66.7)	298 (59.7)	1.335 (0.972–1.888)	0.073	1.382 (0.990–1.929)	0.057	
	Moderate to severe	34 (64.1)	35 (66.0)	0.920 (0.414–2.046)	0.839	0.707 (0.301–1.664)	0.428	
mRS score of 0 to 2 at 90 d^d^	None	358 (79.2)	382 (78.1)	1.067 (0.781–1.458)	0.685	1.070 (0.783–1.463)	0.672	0.231
	Mild	250 (81.4)	219 (73.5)	1.582 (1.076–2.327)	0.020	1.637 (1.109–2.415)	0.013	
	Moderate to severe	42 (79.2)	41 (77.4)	1.118 (0.443–2.817)	0.814	0.815 (0.301–2.206)	0.687	
END within 7 d^d^, ^e^	None	32 (7.1)	37 (7.6)	1.074 (0.657–1.756)	0.775	1.064 (0.649–1.744)	0.807	0.289
	Mild	26 (8.5)	13 (4.4)	0.493 (0.248–0.979)	0.043	0.525 (0.263–1.048)	0.068	
	Moderate to severe	2 (3.8)	3 (5.7)	1.530 (0.245–9.549)	0.649	1.493 (0.238–9.365)	0.669	
SAP within 12 d^d^, ^f^	None	11 (2.4)	11 (2.2)	0.923 (0.396–2.149)	0.852	0.974 (0.417–2.277)	0.952	0.284
	Mild	11 (3.5)	5 (1.7)	0.459 (0.158–1.338)	0.154	0.448 (0.153–1.309)	0.142	
	Moderate to severe	2 (3.8)	1 (1.9)	0.490 (0.043–5.578)	0.566	0.480 (0.042–5.483)	0.555	
Stroke or other vascular events within 90 d^g^	None	1 (0.2)	2 (0.4)	1.852 (0.167–20.496)	0.615	1.916 (0.172–21.324)	0.297	0.165
	Mild	5 (1.6)	2 (0.7)	0.510 (0.093–2.806)	0.439	0.474 (0.086–2.620)	0.392	
	Moderate to severe	1 (1.9)	0 (0)	0.000	0.998	0.000	0.998	
Death within 90 d^g^	None	1 (0.2)	1 (0.2)	0.924 (0.058–14.819)	0.956	1.310 (0.080–21.433)	0.850	0.873
	Mild	3 (1.0)	3 (1.0)	1.031 (0.206–5.147)	0.971	1.148 (0.228–5.785)	0.867	
	Moderate to severe	1 (1.9)	1 (1.9)	1.000 (0.061–16.417)	1.000	0.982 (0.059–16.240)	0.990	

Abbreviations: END, early neurologic deterioration; HR, hazards ratio; mRS, modified Rankin Scale; OR, odds ratio; RIC, remote ischemic conditioning; SAP, stroke‐associated pneumonia.

^a^ Adjusted for unbalanced covariates (sex, smoking consumption, alcohol consumption, hypertension, presumed stroke cause).

^b^ Adjusted for unbalanced covariates (age, sex, smoking consumption, alcohol consumption, hypertension, diabetes).

^c^ mRS scores range from 0 to 6: 0 = no symptoms, 1 = symptoms without clinically significant disability, 2 = slight disability, 3 = moderate disability, 4 = moderately severe disability, 5 = severe disability, and 6 = death.

^d^ Calculated using logistic regression analysis, which showed OR and 95% CI.

^e^ Early neurologic deterioration was defined as an increase between baseline and 7 days of 2 on the NIHSS score, but not a result of cerebral hemorrhage.

^f^ Stroke‐associated pneumonia was defined according to the recommendation from the pneumonia in stroke consensus group.

^g^ Calculated using Cox regression analysis, which showed HR and 95% CI.

Similar results were also found with respect to favorable functional outcome (mRS 0–2) in patients with mildly impaired renal function (adjusted OR = 1.637 [95% CI 1.109–2.415], *p* = 0.013), but not in moderate to severely impaired renal function (adjusted OR = 0.815 [95% CI 0.301–2.206], *p* = 0.687) and normal renal function (adjusted OR = 1.070 [95% CI 0.783–1.463], *p* = 0.672). There was no significant effect of RIC on other secondary outcomes including the occurrence of early neurologic deterioration compared with baseline at 7 days, the occurrence of stroke‐associated pneumonia at 12 days, time from randomization to occurrence of stroke or other vascular events at 90 days, and the occurrence of death within 90 days in any renal function group (Table 3).

**FIGURE 3:**
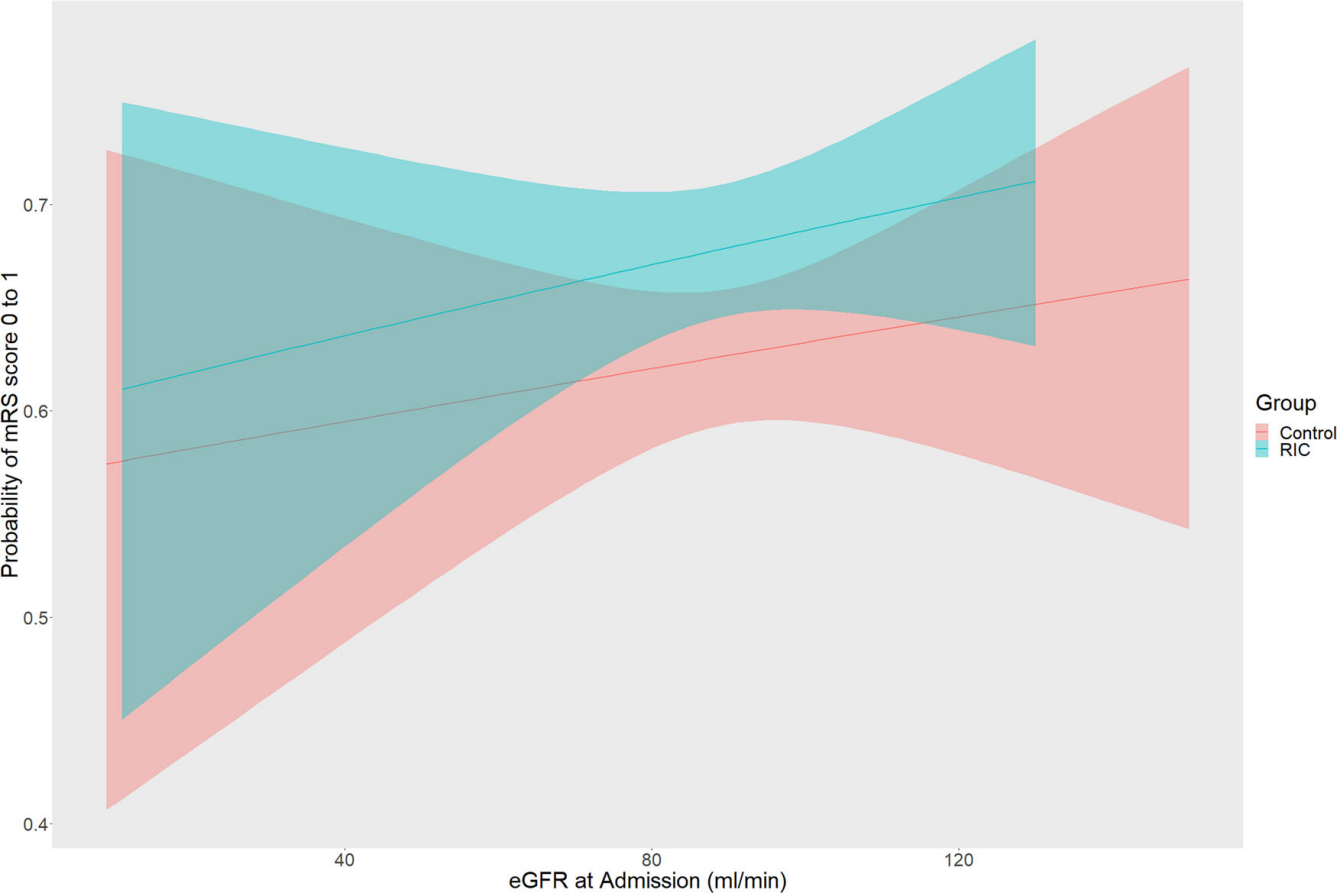
Distribution of modified Rankin Scale (mRS) scores at 90 days between treatment groups.

When eGFR was used as a continuous variable, patients with better renal function were more likely to have excellent functional outcome, and the RIC group was more likely to have excellent functional outcome than the control group with similar renal function (OR = 1.260 [95% CI 1.029–1.544], *p* = 0.026), but this difference decreased with the decline of renal function (Figure 3).

## 4 Discussion

In this post hoc analysis of the RICAMIS trial, our findings indicate that treatment with RIC, as opposed to the control, was associated with an increased probability of achieving an excellent functional outcome at 90 days following symptom onset in patients with normal or mildly impaired renal function. However, it is important to note that the reduction in sample size within the subgroups precluded the attainment of statistical significance.

Consistent with prior epidemiological data (Lv and Zhang 2019), patients with renal impairment were generally older in this study. Notably, only 6.4% of the study population presented with moderate to severe renal dysfunction, and this subgroup exhibited a substantially higher burden of hypertension and diabetes. These observations align with well‐documented evidence regarding the progressive decline in renal function with aging (Kitai et al. 2021), the nephrotoxic effects of hypertension (Hamrahian and Falkner 2017) and diabetes (Thomas et al. 2015), and their established synergistic relationship with stroke risk (Dad and Weiner 2015).

In this secondary analysis, we observed that the therapeutic effect of RIC on the primary endpoint was diminished in patients with moderate to severely impaired renal function, although no interaction effect between renal function and the intervention was detected. Emerging evidence indicates that nephropathy significantly impacts cerebrovascular function, manifesting as impaired cerebral perfusion, dysregulated neurovascular coupling, and compromised vascular integrity (Sedaghat et al. 2016). Moreover, the kidney and brain possess similar microvascular structures and autoregulatory mechanisms, rendering them similarly vulnerable to microvascular dysfunction (Schiller and Covic 2010). These findings imply that renal function may reflect cerebral autoregulation, which has been associated with the efficacy of RIC (Guo et al. 2019). Due to impaired cerebral autoregulation, remodeling of the cerebral vasculature, and reduced cerebral blood flow (CBF) (Castro et al. 2015; Liu et al. 2018), kidney disease has been shown to be associated with a greater neurological deficit following ischemic stroke, worse functional outcome, and greater mortality compared to patients without renal impairment (El Husseini et al. 2017, 2018; Alqahtani et al. 2018), which may weaken the effect of RIC. In addition, renal dysfunction could directly impair the systemic or cerebral protective pathways—including diminished nitric oxide (NO) bioavailability, elevated oxidative stress, and enhanced inflammatory responses—thereby potentially attenuating the therapeutic efficacy of RIC (Cho et al. 2025; Hess et al. 2021). Furthermore, our recent study found that RIC significantly improved the likelihood of excellent functional outcome in non‐diabetic patients, a population with inherently lower risks of renal impairment given the well‐established association between diabetes and kidney dysfunction (Zhang et al. 2023). Taken together, these factors may offset the effect of RIC treatment in patients with moderate to severely impaired renal function.

Interestingly, we found that RIC may be associated with more benefit in patients with mildly impaired renal function, with a higher likelihood of favorable clinical outcomes (mRS 0–1, mRS 0–2), and lower likelihood of END compared to patients with normal renal function. As discussed above, RIC treatment could improve the mild impairment of cerebral autoregulation in this population, but not in patients with moderate to severe impairment of cerebral autoregulation. The improvement by RIC treatment may contribute to the benefit in patients with mildly impaired renal function, compared with patients with normal or moderate to severely impaired renal function.

The strength of the present study was that, to our knowledge, this was the first report to investigate the effect of renal function on the efficacy of RIC based on a large, randomized controlled, multicenter study. However, this study had several limitations. The main one was the sample imbalance between patients with different renal function including a small sample size in patients with moderate to severely impaired renal function, which may have weakened the statistical power as well as the validity of the findings. Second, the analysis population was limited to Chinese individuals, which may affect the generalizability of our results to other ethnic groups. Third, patients who received intravenous thrombolysis and endovascular thrombectomy were excluded from this trial, while RIC combined with reperfusion treatment has potentially neuroprotective effects in acute ischemic stroke patients (Hess et al. 2015; He et al. 2020; Zhao et al. 2018). These limitations would affect the generalizability of this finding. Fourth, due to unknown renal function status before stroke onset, it was difficult to determine whether the eGFR decline was caused by acute kidney injury (AKI) or prior CKD, which may confound the current findings. Fifth, the association of other comorbidities such as several strokes history, myocardial infarction, peripheral artery disease, etc., with RIC efficacy also warrants further investigation. Finally, the nature of the secondary analysis is exploratory and warrants further study.

In conclusion, compared with moderate to severely impaired renal function, patients with normal and mildly impaired renal function have a higher probability of excellent functional outcome at 90 days in the RIC group versus the control group, although no interaction effect of renal function on intervention efficacy was found. This finding should be validated in future studies.

## Author Contributions

**Xiao‐Yi He**: data curation, formal analysis, writing – original draft. **Yu Cui**: formal analysis, writing – original draft. **Jia‐Qi Wang**: data curation, formal analysis. **Lu Wang**: data curation, formal analysis. **Hui‐Sheng Chen**: conceptualization, funding acquisition, project administration, writing – review and editing.

## Conflicts of Interest

The authors declare no conflicts of interest.

## Peer Review

The peer review history for this article is available at https://publons.com/publon/10.1002/brb3.70831.

## Data Availability

The data that support the findings of this study are available from the corresponding author on reasonable request.
